# Occupational Stress, Coping Strategies, and Resilience Among Health and Social Care Professionals Working with Children and Families in Greece: A Cross-Sectional Study

**DOI:** 10.3390/healthcare14142184

**Published:** 2026-07-20

**Authors:** Dimitrios Mimarakis, Philippa Kolokotroni, Eleni Plevriti, Maria Moudatsou, Sofia Koukouli

**Affiliations:** 1 Department of Social Work, Faculty of Health Sciences, Hellenic Mediterranean University, 71410 Heraklion, Greece; moudatsoum@hmu.gr (M.M.); koukouli@hmu.gr (S.K.); 2 Laboratory of Interdisciplinary Approaches for the Enhancement of Quality of Life (QoLab), 71410 Heraklion, Greece; 3 Department of Psychology, Panteion University of Social & Political Sciences, 17671 Athens, Greece; philippakolokot@gmail.com; 4 Panagiotis & Aglaia Kyriakou Children’s Hospital, 11527 Athens, Greece; plevritieleni@gmail.com; 5 School of Medicine National and Kapodistrian, University of Athens, 11527 Athens, Greece

**Keywords:** children, coping strategies, families, Greece, healthcare professionals, occupational stress, resilience

## Abstract

Context: Child-focused healthcare professionals serve as primary providers of both physical and psychosocial care to pediatric patients and their families. Consequently, they are exposed to substantial occupational and emotional challenges in the course of their practice. Objectives: This study examined the levels and multidimensional aspects of occupational stress, coping strategies, and resilience among healthcare professionals providing care to pediatric patients and their families in Greece. Methods: Socio-demographic characteristics, perceived occupational stress, coping strategies (WCQ), and resilience were assessed in a sample of 202 child-focused healthcare (physicians and nurses) and social care professionals (social workers, psychologists, and special education professionals) employed across a range of health and psychosocial services in Greece. Multiple linear regression analyses using the enter method were performed to examine the associations among resilience, occupational stress, and coping strategies while controlling for relevant demographic and occupational variables. Results: Social support was significantly associated with gender. Positive coping was associated with age, work experience, and the WCQ subscales Social Support, Daydreaming, and Avoidance. Avoidance coping was positively associated with profession and Daydreaming. Significant differences were also observed according to age, occupational stress, positive coping, social support, and resilience. Hierarchical multiple regression analysis demonstrated that, positive coping was independently and most strongly associated with resilience (*p* < 0.001), whereas work stress was independently and negatively associated with resilience (*p* = 0.001), with higher perceived stress corresponding to lower levels of resilience. Conclusions: The findings highlight the central role of positive coping in relation to resilience among child-focused healthcare professionals, while demonstrating the negative association between occupational stress and resilience. These results underscore the need for interventions that strengthen effective coping strategies, reduce work-related stress, and enhance resilience among healthcare professionals. They also identify important knowledge gaps and support the need for future research examining occupational stress, its long-term consequences, and the coping mechanisms and resilience among professionals working with children and families.

## 1. Introduction

Child-focused healthcare professionals—including paediatricians, nurses, social workers, psychologists, special educators, and speech and language therapists—provide care for children aged 0–16 years. They serve as the primary providers of both physical and psychosocial support to children and their families. Caring for critically ill or dying children, managing parental anxiety, experiencing secondary traumatic stress, and navigating complex family dynamics represent major sources of occupational stress for healthcare professionals [1]. In addition, providing care for children with suspected maltreatment [2], trauma victims, children exposed to stressful life events [3], developmental disorders, or those at risk of developing such conditions presents additional challenges. These challenges are further complicated by the elevated stress experienced by both children and their families [4]. The emotional burden associated with supporting families facing these challenges may, in turn, affect healthcare professionals’ emotional well-being [5], resilience, and coping strategies. These factors are closely related to occupational stress in demanding paediatric settings.

From a theoretical perspective, the distinctive emotional, ethical, and organizational demands associated with caring for children and their families may influence the experience of occupational stress. They may also affect the coping strategies and resilience processes professionals employ to manage these demands. Consequently, findings derived from studies involving professionals working with adult populations cannot be assumed to generalize to child-focused healthcare settings. From a practical perspective, a better understanding of occupational stress, coping strategies, and resilience within this multidisciplinary workforce may inform the development of targeted interventions, organizational policies, and personnel support initiatives. These approaches may promote professionals’ well-being, prevent burnout, and ultimately improve the quality of care provided to children and their families.

Research by Scanlan and Still [6] demonstrated that job satisfaction among health and social care professionals is strongly associated with both turnover intention and burnout.

To the best of our knowledge, this study is among the first to examine the associations between individual and professional characteristics, occupational stress, coping strategies, and resilience among multidisciplinary child-focused healthcare professionals in Greece. Previous research has primarily focused on individual professional groups, such as nurses or physicians, whereas evidence regarding multidisciplinary child-focused healthcare teams remains limited.

We targeted child-focused healthcare professionals for the following three main reasons: (a) studies involving paediatric professionals remain limited; (b) working with children presents unique emotional and professional challenges that differ from those encountered when caring for adults; and (c) the characteristics of the target population may influence the relationships among occupational stress, coping strategies, and resilience. These relationships were examined in the present study.

### 1.1. Literature Review

#### Job Stress

The World Health Organization (WHO) defines workplace stress as the response individuals experience when work-related demands exceed, or are perceived to exceed, their knowledge and abilities to cope [7]. The literature identifies a broad and often difficult-to-classify range of occupational stressors. Nevertheless, the most frequently reported sources include working conditions [8]. These encompass both tangible factors (e.g., salary, working hours, and physical demands) and intangible factors (e.g., exposure to unpredictable situations and emotionally demanding events). Physicians exhibiting pronounced self-critical and perfectionistic tendencies have been shown to experience lower levels of conscientiousness. Lower conscientiousness subsequently predicts higher levels of burnout through increased emotional exhaustion and depersonalization [9].

Recent evidence indicates that child-focused healthcare professionals frequently experience substantial occupational stress and burnout [10,11]. The association between occupational stress and increased burnout has been consistently documented [12]. Occupational stress has also been associated with poorer physical and psychological health, reduced job satisfaction, and diminished work performance. According to Ginter et al. [13], five principal themes emerged from the perspectives of newly qualified child life professionals regarding workplace stress and burnout. These included the unexpected emergence of burnout, the three dimensions of burnout, a professional culture characterized by burnout, the challenges of self-care, and considerations regarding whether to remain in the child life profession. Similarly, the systematic review conducted by Flynn et al. [14] documented a high prevalence of burnout among paediatric nurses and demonstrated that greater fatigue is associated with increased occupational stress. These findings reinforce the interrelated effects of workload, professional well-being, and patient safety. Job satisfaction also emerged as an important determinant influencing both burnout and the provision of safe clinical care.

### 1.2. Coping Strategies

Coping refers to the cognitive and behavioural efforts individuals employ to manage stressful situations and the emotions they evoke. According to Lazarus and Folkman [15], coping is a dynamic process that depends on individuals’ appraisal of a stressor and the resources available to manage it. The most widely accepted classification distinguishes between problem-focused coping and emotion-focused coping [15]. Problem-focused coping aims to address the source of stress, whereas emotion-focused coping seeks to regulate emotional responses. Subsequent research identified avoidance coping as a distinct category, encompassing strategies such as denial and behavioural disengagement [16,17]. Endler and Parker [18] further classified coping into task-oriented, emotion-oriented, and avoidance-oriented styles. Folkman [19] later introduced meaning-focused coping, emphasizing positive reappraisal and personal values in managing chronic stress. Collectively, these classifications demonstrate that individuals employ a variety of coping strategies depending on the characteristics of the stressful situation [20].

Avoidance coping strategies, such as denying or minimizing stress responses, have been associated with higher levels of compassion fatigue and burnout. They have also been linked to lower levels of compassion satisfaction [11]. Among child-focused healthcare professionals, Ginter et al. [13] reported that coping frequently involves establishing clear boundaries with family members and friends regarding when, where, and how work-related experiences are discussed. Nevertheless, Hoelscher and Ravert [10] found that child-focused healthcare professionals continue to experience difficulties maintaining effective work–life boundaries, although the specific types of boundaries were not described.

Similarly, Hurley et al. [21] identified four major coping themes among nurses caring for children with life-limiting conditions. These included an evolving sense of duty, reflecting increasing emotional resilience through repeated exposure to death and suffering; establishing boundaries as a means of protecting emotional well-being; drawing strength from interpersonal connections, highlighting the importance of teamwork and social support; and faith as a foundation of care, emphasizing the role of spirituality in sustaining professional practice.

### 1.3. Resilience

Healthcare resilience is increasingly recognized as a fundamental determinant of high-quality healthcare delivery. It has been defined as the proactive capacity of healthcare organizations, units, teams, and individual professionals to anticipate, adapt to, and effectively respond to change and everyday challenges [22]. Rather than merely resisting change, healthcare resilience supports the delivery of safe and high-quality care. In other words, resilience represents a dynamic, multilevel adaptive capacity that enables healthcare systems and professionals to respond effectively to routine challenges while maintaining the quality of care provided [22]. Healthcare resilience is supported by the adaptive capacity of healthcare systems through the mobilization of both internal resources (e.g., sense-making and professional experience) and external resources (e.g., colleagues, professional networks, and regulatory frameworks). These resources allow professionals to adjust their everyday practice. Such adaptations may include modifications to care processes in response to fluctuating demands or time constraints. These adaptations facilitate the successful management of complex clinical situations while maintaining high standards of care [23].

The review conducted by Sheikhrabori et al. [24], which examined the individual and contextual factors contributing to resilience among healthcare professionals, identified effective coping as one of the principal characteristics associated with resilience.

Resilience has consistently been identified as a key protective factor against professional burnout. It enhances healthcare professionals’ self-confidence, self-regulation, and ability to manage occupational stress effectively. Evidence further suggests that empathy and self-compassion, together with resilience, contribute to burnout prevention [4,25,26]. Healthcare professionals with higher resilience also demonstrate a greater capacity to cope with occupational stress. Resilience in healthcare is conceptualized as a dynamic, multilevel adaptive capacity supported by both internal and external resources [22]. Internal resources, including cognitive appraisal processes and adaptive coping strategies, facilitate more constructive interpretations of stressful situations. These processes enhance individuals’ capacity to cope effectively with occupational demands. Conversely, maintaining adaptive functioning in highly demanding clinical environments also depends on external resources, including support from colleagues and professional networks. However, these adaptive systems may become compromised following prolonged exposure to high occupational demands. The subjective perception of persistent work-related stress may gradually erode resilience by reducing the availability and effectiveness of both psychological and social resources. The literature consistently demonstrates an inverse relationship between resilience and perceived occupational stress. Evidence from systematic reviews involving healthcare professionals indicates that nurses experiencing higher levels of occupational stress exhibit lower resilience [27]. This reflects a diminished capacity to recover and adapt in highly demanding work environments. This inverse association highlights resilience as a protective psychological resource. However, this resource may be weakened by sustained emotional and organizational pressures, particularly during prolonged exposure to occupational strain. Despite the growing international literature on occupational stress, coping strategies, and resilience among healthcare professionals, important knowledge gaps remain, particularly in paediatric care settings and multidisciplinary healthcare teams. Most previous studies have focused on specific professional groups, such as physicians or nurses, whereas integrated health and social care teams working with children and families have received comparatively little attention. Furthermore, research within the Greek context remains limited, particularly regarding the simultaneous investigation of occupational stress, coping strategies, and resilience among professionals providing child-focused healthcare services.

Accordingly, the present study aimed to examine the associations between occupational stress, coping strategies, demographic and professional characteristics, and resilience among healthcare and social care professionals working with children and families in Greece. Specifically, the study examined whether coping strategies and perceived occupational stress were associated with resilience after accounting for demographic and occupational characteristics.

Based on previous literature, the following hypotheses were formulated:

- Higher perceived occupational stress will be associated with lower levels of resilience.
- Adaptive coping strategies (positive coping and social support) will be positively associated with resilience, whereas avoidance coping will show a negative association with resilience.
- Coping strategies and perceived occupational stress will account for additional variance in resilience beyond demographic and occupational characteristics.

## 2. Materials and Methods

### 2.1. Study Design

This study employed a quantitative, cross-sectional design involving health and social care professionals working with children, adolescents, and their families. A non-probability purposive sampling strategy was used, whereby individuals meeting the eligibility criteria were intentionally selected to ensure inclusion of professionals with relevant clinical and psychosocial experience.

### 2.2. Participants and Data Collection

A total of 202 Greek professionals employed in a range of health and social care settings serving children and adolescents participated in the study. Participants were eligible if they (1) were health or social care professionals, (2) were currently employed in services providing care to children and adolescents in Greece, and (3) were aged 18 years or older.

Data were collected using a structured questionnaire consisting of four sections, administered via Google Forms and made available online for a 12-month period. To minimize sampling bias, the questionnaire was distributed across multiple institutions and geographic regions in Greece, targeting professionals from diverse disciplines including medicine, nursing, social work, psychology, and special education. Distribution was carried out through professional networks, primarily via email mailing lists of officially recognized professional associations and organizations. A substantial proportion of participants were recruited through the second largest pediatric hospital in Greece (“Pan. & Aglaias Kyriakou” Children’s Hospital), through bulletins of the Paediatric Association of Athens and the Hellenic Society of Pediatric Nursing, and through social media platforms.

A total of 202 complete responses were included in the analysis. Cases with missing data on any study variable were excluded using listwise deletion in IBM SPSS Statistics 23.0, and all analyses were conducted on complete cases. No a priori power analysis was performed prior to data collection; however, the achieved sample size (*n* = 202) is considered adequate for multiple linear regression models according to general methodological recommendations for detecting small-to-moderate effects. Participation was voluntary, and informed consent was obtained from all participants.

### 2.3. Measures

#### 2.3.1. Socio-Demographic and Occupational Characteristics

Participants provided self-reported socio-demographic information (e.g., gender, age, marital status, education level) and occupational characteristics (e.g., profession, years of experience, employment status, workplace setting, and managerial responsibility).

#### 2.3.2. Job Stress Measure (JSM)

Occupational stress was assessed using the Job Stress Measure (JSM), a 16-item self-report instrument designed to evaluate perceived stress arising from specific and identifiable workplace stressors. The scale was originally developed by Judge, Boudreau, and Bretz [28] and targets key occupational dimensions such as workload, time pressure, role ambiguity, organizational politics, and career uncertainty.

Items are rated on a 5-point Likert scale indicating the extent to which each job-related factor is perceived as stressful. Higher scores indicate greater perceived occupational stress. The Greek validation of the instrument was performed by Sakketou et al. [29], demonstrating good internal consistency (Cronbach’s α = 0.868).

#### 2.3.3. Ways of Coping Questionnaire (WCQ)

Coping strategies were assessed using the Greek validated version of the Ways of Coping Questionnaire (WCQ) [30]. Originally developed by Folkman and Lazarus, the WCQ measures cognitive and behavioral responses used to manage the internal and external demands of stressful situations [31].

The Greek version consists of 38 items rated on a 4-point Likert scale ranging from 0 (“never”) to 3 (“often”) [32]. For the present study, the following subscales were included: positive coping, social support, avoidance, daydreaming and assertive problem solving. Scores are not aggregated into a global index; instead, mean scores are calculated for each factor, with higher values indicating more frequent use of the corresponding coping strategy [32]. Cronbach’s alpha for the five factors was found to be relatively high, ranging from 0.60 to 0.79 [32].

#### 2.3.4. Connor–Davidson Resilience Scale (CD-RISC)

Resilience was measured using the Connor–Davidson Resilience Scale (CD-RISC) [33], a widely used instrument assessing the ability to cope with adversity, stress, and change. The scale consists of 25 items rated on a Likert-type scale, with higher scores indicating greater resilience.

The Greek adaptation of the Connor–Davidson Resilience Scale was developed and psychometrically validated by Tsigkaropoulou et al., demonstrating strong psychometric properties (Cronbach’s alpha for the total scale generally exceeding 0.90) [34].

#### 2.3.5. Ethical Considerations

The study was conducted in accordance with the ethical principles outlined in the Declaration of Helsinki for research involving human participants. Participation was voluntary, and all participants were fully informed about the aims and procedures of the study prior to data collection. Informed consent was obtained from all participants before completion of the questionnaire. Participants were assured of the anonymity and confidentiality of their responses, and no personally identifiable information was collected.

Data were stored securely and used exclusively for research purposes. Participants were informed that they could withdraw from the study at any time without any consequence. Given the anonymous and non-invasive nature of the study design, no additional risks to participants were anticipated.

The study protocol was reviewed and approved by the Scientific Council of “Panagiotis & Aglaia Kyriakou” Children’s Hospital of Athens, Greece (14120/9 August 2021), as well as from the Board of Directors of the same Hospital (15060/31 August 2021).

### 2.4. Statistical Methods

Prior to the main analyses, the distribution of continuous variables was assessed using histograms, normal Q-Q plots, skewness and kurtosis statistics, and Shapiro–Wilk tests. The observed distributions demonstrated acceptable approximation to normality, supporting the use of parametric statistical analyses. Potential outliers were assessed through visual inspection of boxplots. No extreme observations requiring exclusion were identified, and all cases were retained for analysis.

Continuous variables, such as questionnaire scores, were presented as means (M) and standard deviations (SD), whereas categorical variables were reported as frequencies (*n*) and percentages (%). Skewness and kurtosis indices, along with the Kolmogorov–Smirnov and Shapiro–Wilk tests, as well as visual inspection of histograms and Q–Q plots, were used to assess the normality of continuous variables. Based on these assessments, the use of parametric statistical methods was considered appropriate. The internal consistency of the research instruments was evaluated using Cronbach’s alpha coefficient.

Categorical variables (including profession, employment status, work environment and work experience) were recoded into indicator (dummy) variables prior to inclusion in the regression analyses. This approach is consistent with standard recommendations for incorporating categorical predictors into multiple linear regression models and facilitates interpretation of regression coefficients relative to the reference category. Reference-category coding was applied, with the largest category selected as the reference group for interpretation purposes.

Associations between categorical variables were analyzed using chi-square (χ^2^) tests, with effect sizes estimated using Cramer’s V. Differences between groups were examined using independent-samples *t* tests for two-group comparisons and one-way analyses of variance (ANOVA) for comparisons involving more than two groups. When significant differences were identified, post hoc analyses were conducted using Tukey’s HSD test. The assumption of homogeneity of variances was assessed using Levene’s test. To enhance the stability and interpretability of the analyses, selected demographic and occupational variables (e.g., age, profession, and work setting) were recoded into broader categories. Pearson correlation coefficients (r) were calculated to examine linear relationships between continuous variables.

Multiple linear regression analyses were conducted using the enter method to examine the relationships among resilience (CD-RISC-25), work stress (JSM), and coping strategies (Ways of Coping Questionnaire subscales), while accounting statistically for relevant demographic and occupational characteristics. In addition, hierarchical multiple regression analyses were performed to assess the incremental contribution of work stress and coping strategies to resilience, with variables entered in theoretically informed blocks. The order of variable entry was theoretically informed by stress, coping, and resilience frameworks. Specifically, the Transactional Model of Stress and Coping [15] posits that resilience is related to individuals’ appraisal and coping processes in response to stress, suggesting that demographic and contextual factors represent more distal influences, whereas coping strategies and perceived stress are more proximal factors associated with resilience. In addition, resilience theory emphasizes the role of protective factors and stress exposure in adaptive functioning [35]. Based on these perspectives, variables were entered hierarchically as follows: demographic and occupational characteristics (work experience, profession, employment status, and work environment) in Step 1, coping strategies (positive coping, social support, and avoidance coping) in Step 2, and perceived work stress in Step 3. All regression models were examined for agreement with key assumptions, including linearity, normality of residuals, homoscedasticity, independence of errors (assessed using the Durbin–Watson statistic), and absence of multicollinearity (assessed using VIF and tolerance values). No substantial violations of these assumptions were observed.

All statistical analyses were performed using IBM SPSS Statistics (Version 23.0), and the level of statistical significance was set at α = 0.05.

## 3. Results

### 3.1. Participants’ Profile and Chi-Square Analyses

Table 1 presents the demographic and professional characteristics of the study sample. A total of 202 healthcare and related professionals participated in the study, the majority of whom were female (*n* = 149, 73.8%). The largest age group was 35–54 years (*n* = 114, 56.4%), and most participants were married (*n* = 131, 64.9%). Over half of the sample (*n* = 104, 51.4%) held postgraduate qualifications (Master’s or doctoral degrees), while 49.0% (*n* = 99) reported more than 16 years of professional experience.

The sample consisted of physicians (including consultants and residents), nurses, and psychosocial professionals, categorized for analytical purposes into three groups: medical (45.0%), nursing (23.8%), and psychosocial (31.2%). Regarding employment characteristics, 46.5% of participants were permanent employees, 52.0% worked in hospital settings, and 27.2% held leadership positions. Overall, the sample comprised predominantly mid-career, highly educated professionals working mainly in hospital environments.

Chi-square analyses were conducted to examine associations between professional characteristics and key study variables. No statistically significant association was observed between gender and professional category (medical, nursing, psychosocial), χ^2^(2) = 0.80, *p* = 0.671, indicating a similar distribution of professional groups across males and females. A statistically significant association was found between professional category and employment status, χ^2^(4) = 62.30, *p* < 0.001, Cramer’s V = 0.393, indicating a moderate-to-large strength of association. Physicians were more likely to be self-employed, nurses predominantly held permanent positions, and residents were mainly contract-based employees.

A strong and statistically significant association was also identified between professional category and working setting, χ^2^(4) = 146.44, *p* < 0.001, Cramer’s V = 0.602, indicating a large strength of association. Nurses were primarily hospital-based, psychosocial professionals were mainly employed in psychosocial/educational services, while physicians were distributed between hospital and community settings. Finally, a statistically significant association was observed between professional category and educational level, χ^2^(6) = 40.15, *p* < 0.001, Cramer’s V = 0.315, indicating a moderate strength of association. Physicians and psychosocial professionals were more likely to hold postgraduate qualifications, whereas nurses were more frequently concentrated at lower educational levels.

### 3.2. Descriptive Statistics and Internal Consistency of Study Variables

Table 2 presents the descriptive statistics and internal consistency coefficients for all study variables (*n* = 202). Overall, participants reported a moderate level of work stress (M = 49.95, SD = 11.68), while resilience scores were relatively high (M = 68.32, SD = 13.10). Regarding coping strategies, mean scores indicated moderate use of positive coping (M = 2.10, SD = 0.47) and social support (M = 2.07, SD = 0.55), whereas avoidance (M = 1.60, SD = 0.50) and daydreaming (M = 1.51, SD = 0.64) were reported at lower levels. All variables demonstrated acceptable distributions, with skewness and kurtosis values falling within generally acceptable limits (approximately ±1), suggesting no major deviations from normality.

Internal consistency analyses indicated good to excellent reliability for most scales. Specifically, the Job Stress Scale (JSM; 16 items) demonstrated very high reliability (Cronbach’s α = 0.891), as did the CD-RISC resilience scale (25 items; α = 0.893). Among the coping subscales, reliability was good for positive coping (α = 0.830) and daydreaming (α = 0.813), and acceptable for social support (α = 0.733). The avoidance subscale showed marginal reliability (α = 0.684), suggesting cautious interpretation in subsequent analyses. Due to low internal consistency (Cronbach’s α = 0.540), the assertiveness subscale was excluded from further analyses, as it did not meet the minimum acceptable reliability threshold for research use in the present sample.

### 3.3. Associations Between Demographic Factors, Work Stress, Resilience, and Coping Strategies

A series of independent-samples *t*-tests and one-way ANOVAs were conducted to explore whether study variables differed across demographic and professional characteristics. Overall, most psychological variables showed no meaningful variation across socio-demographic groups. No gender differences were found in work stress, resilience, positive coping, daydreaming, or avoidance coping. However, women reported significantly higher perceived social support than men, t(200) = −2.94, *p* = 0.004. Age was related to two variables. Significant differences were found for resilience, F(2, 199) = 3.53, *p* = 0.031, and positive coping, F(2, 199) = 7.56, *p* = 0.001. Younger participants (under 34 years) reported lower resilience than those aged 35–54 years (*p* = 0.034), and also lower positive coping compared to both older groups (*p* ≤ 0.004). No other age-related differences were observed.

In terms of professional category, most variables did not differ significantly, with the exception of avoidance coping, F(2, 199) = 5.72, *p* = 0.004. Nurses reported higher levels of avoidance coping compared to psychosocial professionals (*p* = 0.003). Work setting, educational level, and marital status were not associated with any of the study variables.

Finally, work experience showed a more specific pattern: it was associated with both work stress, F(2, 199) = 3.21, *p* = 0.043, and positive coping, F(2, 199) = 4.87, *p* = 0.009. Participants with moderate experience reported higher work stress than those with low experience (*p* = 0.019), while those with higher experience reported greater positive coping than both lower-experience groups (*p* ≤ 0.036). No differences were found for resilience or the remaining coping strategies.

Pearson correlations were used to explore how the main study variables relate to each other. Overall, resilience showed the most consistent associations with other psychological constructs. Work stress showed a small, but significant negative relationship with resilience, r = −0.21, *p* = 0.003 meaning that higher resilience was associated with lower perceived stress at work. At the same time, work stress was not meaningfully related to coping strategies or social support in this sample.

Resilience, on the other hand, was clearly related to more adaptive psychological resources. It showed a strong positive relationship with positive coping, r = 0.735, *p* < 0.001, and a moderate positive relationship with social support, r = 0.384, *p* < 0.001. This pattern suggests that more resilient individuals tend to rely more on constructive coping strategies and also feel more supported socially.

Positive coping was also positively associated with social support, r = 0.462, *p* < 0.001, meaning that people who use more adaptive coping strategies also tend to perceive stronger support from others. Interestingly, positive coping also showed smaller but still significant links with both daydreaming, r = 0.335, *p* < 0.001, and avoidance coping, r = 0.232, *p* = 0.001. Finally, daydreaming and avoidance coping were strongly connected to each other, r = 0.603, *p* < 0.001, suggesting that these two disengagement-oriented coping styles frequently co-occur.

### 3.4. Hierarchical Multiple Regression Analysis (Resilience as Dependent Variable)

A hierarchical multiple regression analysis was conducted to examine the associations of demographic and occupational characteristics, coping strategies, and work stress with resilience (Table 3). Variables were entered in theoretically informed sequential blocks using the enter method. In Step 1, dummy-coded demographic and occupational independent variables were entered into the model. The model was not statistically significant, F(8193) = 1.67, *p* = 0.107, explaining 6.5% of the variance in resilience (R^2^ = 0.065, Adjusted R^2^ = 0.026). None of the demographic or occupational contrasts showed significant associations with resilience.

In Step 2, coping variables (positive coping, social support, and avoidance) were added. This step resulted in a substantial and statistically significant improvement in model fit, F(11, 190) = 21.75, *p* < 0.001, with explained variance increasing to 55.7% (R^2^ = 0.557, Adjusted R^2^ = 0.532). Positive coping was strongly positively associated with resilience (β = 0.722, *p* < 0.001), whereas neither social support nor avoidance coping reached statistical significance.

In Step 3, work stress was entered into the model. The final model remained statistically significant, F(12, 189) = 21.78, *p* < 0.001, explaining 58.0% of the variance in resilience (R^2^ = 0.580, Adjusted R^2^ = 0.554). Positive coping showed the strongest positive association with resilience in the final model (β = 0.710, *p* < 0.001), whereas work stress was significantly negatively associated with resilience (β = −0.159, *p* = 0.002), indicating that higher levels of perceived work stress were associated with lower resilience. Social support, avoidance coping, and all occupational variables remained non-significant (Table 3 and Table 4).

All assumptions for multiple regression were adequately met. No problematic multicollinearity was observed (Tolerance = 0.220–0.914; VIF = 1.09–4.54). The Durbin–Watson statistic indicated independence of residuals (DW = 1.87). Examination of standardized residuals (−2.86 to 2.30) suggested no serious outliers, and residual distribution indicated acceptable normality and homoscedasticity.

## 4. Discussion

To the best of our knowledge, this study is among the first in Greece to examine the association between demographic and professional characteristics, occupational stress, coping strategies, and resilience. The study focuses specifically on child-focused healthcare professionals. This contribution is important given the growing recognition that resilience is not a static trait but a dynamic process involving continuous interactions between individual resources and contextual work demands.

Overall, child-focused healthcare professionals reported a moderate level of work-related stress. Rather than indicating uniformly high distress, this level may reflect a balance between the emotionally demanding nature of the profession and the development of adaptive professional competencies over time. A significant association was found between years of experience and work stress. Mid-experience professionals reported higher stress compared to their less experienced counterparts. This pattern suggests a non-linear relationship between career stage and stress, where stress may increase after initial adjustment but before full professional consolidation. Consistent with these results, Izdebski et al. [36] reported that mid-career professionals (6–10 years of service) were 2.6 times more likely to experience burnout compared to their early-career or senior counterparts. Similar trends have been observed in child protection and welfare settings, where individuals with more than one year of experience exhibited higher burnout levels [37,38].

These findings may be explained by evolving role expectations across career stages. Early-career professionals may be more focused on learning and skill acquisition, which can buffer stress perceptions. In contrast, mid-career professionals often experience a transition period characterized by increased responsibility, higher expectations, and reduced external supervision. These factors may amplify perceived workload and emotional strain. At later stages, greater professional autonomy and mastery may restore a sense of control, thereby reducing stress. From a broader occupational perspective, similar patterns have been reported across healthcare professions. However, the magnitude and direction of stress vary depending on organizational structure, staffing levels, and professional autonomy. These findings suggest that occupational stress is shaped not only by experience but also by systemic working conditions. This interpretation aligns with resilience frameworks in healthcare [22]. These frameworks emphasize that prolonged exposure to job demands can gradually exhaust internal and external coping resources, thereby reducing adaptive capacity over time.

Regarding coping strategies, participants demonstrated moderate use of positive coping and social support, while avoidance and daydreaming were less frequently used. This pattern suggests a generally adaptive coping profile within this professional sample, although the presence of avoidance strategies indicates residual vulnerability under stress. Gender differences were observed, with women reporting higher levels of perceived social support. This may reflect both differences in help-seeking behavior and broader socialization processes. Consistent with this finding, Kneavel [39] reported that females experience greater quality social support. According to Taylor’s Tend and Befriend theory [40], females are more likely to adopt a “tending and befriending” stress response strategy. This strategy involves seeking and engaging with social support networks during stressful situations. This increased reliance on social support networks may con-tribute to the development of larger support systems and a higher perceived quality of social support.

Age and work experience were positively associated with adaptive coping, with younger professionals reporting lower levels of positive coping. This suggests that coping strategies may develop and consolidate over time through accumulated exposure to workplace stressors and professional challenges. Age-related differences in coping have been widely documented. The study by Chen et al. [41] demonstrates that younger adults are more likely to use less adaptive coping strategies. Similarly, Beier [42] found that age is positively associated with effective coping and personal accomplishment. Moreover, professionals with greater work experience reported more positive coping, suggesting that greater experience in healthcare environments is associated with more adaptive stress-management strategies. This is also supported by Huang et al. [43], which highlights the role of both gender and professional experience in shaping coping styles. Specifically, women and participants with greater work experience were more likely to adopt positive coping strategies compared to men and those with less work experience.

The observed positive relationship between adaptive coping and social support further highlights the interdependence of internal and external resilience resources. Individuals who actively engage in problem-focused coping may be more likely to seek and maintain supportive relationships. Conversely, those perceiving stronger support systems may feel more capable of employing adaptive strategies. Interestingly, the weaker associations between positive coping and maladaptive strategies such as avoidance and daydreaming suggest that coping processes are not strictly dichotomous but may coexist within individuals depending on situational demands.

Differences in avoidance coping across professional groups also warrant attention. Nurses reported higher levels of avoidance coping compared with psychosocial professionals, suggesting variation in coping responses across occupational roles. This may reflect differences in job structure, emotional demands, and exposure to acute clinical situations. For instance, nursing roles often involve high-intensity, time-pressured decision-making and continuous patient contact. These demands may increase reliance on short-term disengagement strategies as a form of psychological protection. In contrast, psychosocial professionals may employ more reflective or emotionally processing-oriented coping styles. This may be related to the nature of their training and professional role expectations. Previous research similarly indicates that avoidance coping is frequently observed among surgical nurses [44], suggesting that occupational context plays a key role in shaping coping behaviors.

Resilience levels among child-focused healthcare professionals were relatively high, suggesting the presence of substantial psychological adaptation despite occupational stressors. Significant age differences were observed in resilience, with younger professionals reporting lower resilience. This may reflect limited cumulative exposure to clinical stressors and fewer opportunities to develop advanced coping repertoires. Similar findings have been reported in healthcare settings in Taiwan, where younger professionals demonstrated lower resilience during the post-pandemic period [45]. Age-related increases in resilience may be explained by accumulated professional experience, improved emotional regulation, and enhanced confidence in managing complex clinical situations [45,46].

Work stress showed a small but significant negative association with resilience, indicating that higher stress is linked to reduced adaptive capacity. Rather than indicating a simple inverse relationship, this pattern suggests a dynamic association between resilience and stress. Resilience may be related to lower levels of stress, while sustained exposure to stress may negatively affect resilience. Resilience is widely recognized as being associated with lower burnout and with more effective coping and emotional regulation. The findings of Pedro Ferreira et al. [47] further support this relationship. Increases in resilience are associated with reductions in emotional exhaustion and depersonalization, alongside improvements in personal accomplishment.

Importantly, resilience was strongly associated with positive coping and moderately associated with social support. These findings reinforce the view that resilience emerges from the interaction between internal psychological resources and external relational systems. Individuals with higher resilience tend to report more effective coping strategies and stronger perceived support networks, which are associated with a greater capacity to manage occupational stress.

The hierarchical regression analysis further indicated that demographic and occupational variables alone explained a limited proportion of the variance in resilience and were not significantly associated with resilience scores within the model. In contrast, the substantial increase in explained variance following the inclusion of coping strategies suggests that coping-related variables showed stronger statistical associations with resilience. Positive coping and social support were positively associated with resilience at the bivariate level, whereas avoidance coping showed a less favorable association pattern. However, after controlling for the other variables included in the mode, only positive coping remained significantly associated with resilience in the final model. The additional contribution of work stress to the model indicates that perceived occupational stress was independently and negatively associated with resilience, although the cross-sectional design does not allow conclusions regarding the directionality of this relationship.

Overall, the final model explains a considerable proportion of variance in resilience. These findings underscore the importance of targeting adaptive coping strategies in interventions aimed at enhancing resilience among child-focused healthcare professionals. These findings align with broader evidence in the healthcare literature, which consistently shows that psychological resources such as coping strategies and social support enhance resilience, whereas chronic occupational stress and burnout reduce it [27]. The systematic review by Yu et al. [27] similarly indicates that coping skills, social support, self-efficacy, job satisfaction, job retention, and well-being are positively associated with nurse resilience. In contrast, work-related stress and burnout are negatively associated with resilience. The negative relationship between stress and resilience is also well established across multiple studies [48,49,50,51,52,53,54,55,56,57], suggesting a robust and consistent pattern across healthcare settings.

Taken together, these findings support the conceptualization of resilience as a modifiable and intervention-sensitive construct. This has important implications for healthcare organizations. Interventions should strengthen coping resources and social support systems while simultaneously addressing structural stressors in the workplace.

Although the present findings provide important insights into factors associated with resilience among child-focused healthcare professionals, several potentially influential variables were not assessed and should be considered when interpreting the results. Personal resources such as family support, caregiving responsibilities, personality characteristics, previous experiences of burnout, and mental health status may substantially influence professionals’ perceptions of occupational stress. These factors may also affect the development of coping strategies. Likewise, workplace-related factors including staffing adequacy, workload, shift patterns, leadership style, organizational climate, access to supervision, and institutional support were not examined. These factors have been consistently identified as important potential relevant variables of resilience and occupational well-being in healthcare settings. Future studies incorporating these individual and organizational variables could provide a more comprehensive understanding of the mechanisms underlying resilience and help identify modifiable targets for intervention. Furthermore, the findings should be interpreted within the Greek sociocultural context. Strong family ties, collectivistic values, and reliance on informal social support networks may shape coping behaviors and perceptions of resilience differently than in countries with more individualistic social structures. At the same time, the prolonged impact of economic austerity and ongoing workforce pressures within the Greek healthcare system may contribute to occupational stress in ways that differ from other healthcare contexts. Consequently, caution is warranted when generalizing these findings to other countries. Cultural norms, healthcare system organization, and workplace environments may influence the relationships between stress, coping, and resilience.

## 5. Strengths and Limitations of This Study

### 5.1. Strengths

To our knowledge, this is one of the few studies in Greece to examine the associations among occupational stress, coping strategies, resilience, and individual and professional characteristics in health and social care professionals working with children and families. A key strength is the inclusion of a multidisciplinary sample comprising physicians, nurses, social workers, psychologists, and special education professionals, a group that has received limited attention in previous research. Unlike most studies focusing on a single professional category, participants were recruited from hospital, clinical, community, and educational settings, enhancing the diversity and relevance of the findings. Although women and physicians were overrepresented, this reflects the characteristics of the healthcare personnel, while the categories of nurses and psychosocial professionals are nearly counterbalanced and numerically comparable to the predominant physician group. Furthermore, the hierarchical multiple regression model explained approximately 60% of the variance in resilience, supporting the robustness of the analytical approach.

### 5.2. Limitations

Several limitations should be acknowledged. Although no a priori power analysis was performed, the final sample size was adequate for multiple regression analyses. The use of an online questionnaire precluded calculation of the response rate and the number of professionals who declined research participation. In addition, the cross-sectional design does not allow causal inferences, and the use of self-report measures may have introduced recall, social desirability, and common method bias, potentially inflating the observed associations. The relatively strong association between positive coping and resilience may partly reflect shared method variance, although these constructs remain conceptually distinct. Finally, the large number of univariate analyses increases the possibility of Type I error; therefore, the findings should be interpreted cautiously and confirmed in future longitudinal and hypothesis-driven studies. In addition, interaction effects among the study variables were not examined, as the study focused on their direct associations; future research should explore potential moderation effects.

## 6. Conclusions

The findings of the present study identify key factors associated with occupational stress, coping, and resilience among child-focused healthcare professionals. Positive coping showed the strongest association with resilience, whereas occupational stress was negatively associated with resilience, emphasizing the importance of strengthening adaptive coping mechanisms within pediatric health and social care settings.

From a policy perspective, these findings support the implementation of comprehensive personnel well-being strategies across health and psychosocial care services. Such strategies may include routine assessment of occupational stress and resilience, evidence-based stress management and resilience training, structured education in adaptive coping skills, and regular reflective clinical supervision. Health and psychosocial care institutions should also ensure timely access to confidential psychological support services and foster supportive multidisciplinary team cultures that encourage peer support, collaboration, and open communication. Furthermore, integrating resilience-related competencies—including emotional intelligence, empathy, and self-care—into undergraduate education, continuing professional development, and leadership training may strengthen professionals’ capacity to manage occupational demands. At the organizational level, policies promoting adequate staffing, manageable workloads, protected time for supervision and professional reflection, and psychologically safe work environments may further contribute to reducing occupational stress and sustaining personnel resilience. Collectively, these measures have the potential to enhance staff well-being, improve retention, and support the delivery of high-quality pediatric and family-centered care. Future longitudinal and intervention studies are warranted to assess these policy initiatives and to further elucidate the mechanisms underlying the relationships between occupational stress, coping, and resilience.

## Figures and Tables

**Table 1:** Demographic and Professional Characteristics of the Health and Social Care Personnel.

Gender	*n*	(%)
Males	53	(26.2%)
Females	149	(73.8%)
Age		
<34	41	(20.3%)
35–54	114	(56.4%)
>55	47	(23.3%)
Marital Status		
Single	56	(27.7%)
Married	131	(64.9%)
Divorced	15	(7.4%)
Educational Level		
Secondary/University	98	(48.6%)
Master of Science/ Doctor of Philosophy	104	(51.4%)
Profession		
Physicians	91	(45.0%)
Nurses	48	(23.8%)
Psychosocial Professionals	63	(31.2%)
Professional Experience (in years)		
0–5	36	(17.9%)
6–15	67	(33.2%)
>16	99	(49.0%)
Employment Status		
Permanent	94	(46.5%)
Contract auxiliary	55	(27.2%)
Self-employed	53	(26.2%)
Working Place		
Hospital Setting	105	(52.0%)
Community/Outpatient Setting	51	(25.2%)
Psychosocial & Educational Services	46	(22.8%)
Head of the Department		
Yes	55	(27.2%)
No	147	(72.8%)

**Table 2:** Descriptive Statistics of Study Variables (*n* = 202).

Scale/Subscale	Mean (M)	SD	Min	Max	Skewness	Kurtosis	Cronbach’s α
Job Stress (JSM)	49.95	11.68	19	80	−0.20	−0.10	0.891
Resilience (CD-RISC)	68.32	13.10	32	100	−0.18	−0.09	0.893
Positive coping (WCQ)	2.10	0.47	0.73	3.00	−0.16	−0.42	0.830
Social support (WCQ)	2.07	0.55	0.83	3.00	−0.31	−0.65	0.733
Daydreaming (WCQ)	1.51	0.64	0.11	3.00	0.06	−0.54	0.813
Avoidance (WCQ)	1.60	0.50	0.13	2.88	−0.07	0.15	0.684

**Note**. (M = mean; SD = standard deviation; Cronbach’s alpha values indicate internal consistency reliability. Values ≥ 0.70 are considered acceptable, while values < 0.70 indicate limited reliability (use with caution).

**Table 3:** Hierarchical Regression Model Fit for Resilience.

Model	Variables Entered	R^2^	ΔR^2^	F	p
*Step 1*	Dummy-coded demographic and occupational variables	0.065	—	1.674	0.107
*Step 2*	+Coping strategies	0.557	0.492 ***	21.746	<0.001
*Step 3*	+Work stress	0.580	0.023 **	21.777	<0.001

**Note1**. Step 1 included work experience (0–5 years, 6–15 years; reference category: >16 years), profession (Nurse, Psychosocial; reference category: Medical), employment status (Contract, Self-employment; reference category: Permanent), and work environment (Community, Psychosocial; reference category: Hospital). Step 2 included positive coping, social support, and avoidance coping. Step 3 included work stress. ΔR^2^ represents the change in explained variance after the addition of each block. **Note2**. ** *p* < 0.01. *** *p* < 0.001.

**Table 4:** Final Hierarchical Regression Model with Resilience as the Dependent Variable (Step 3).

Predictor	B	SE	β	95% CI	*p*
Work experience (reference: >16 years)					
0–5 years	0.179	2.077	0.005	[−3.919, 4.276]	0.932
6–15 years	0.790	1.589	0.028	[−2.345, 3.925]	0.620
Profession (reference: Medical)					
Nurse	0.597	1.830	0.019	[−3.013, 4.208]	0.744
Psychosocial	1.198	2.214	0.042	[−3.170, 5.567]	0.589
Employment status (reference: Permanent)					
Contract	−0.792	1.778	−0.027	[−4.300, 2.716]	0.656
Self-employment	−1.058	2.971	−0.036	[−6.920, 4.803]	0.722
Work environment (reference: Hospital)					
Community	1.329	3.021	0.044	[−4.630, 7.287]	0.661
Psychosocial	−2.098	2.198	−0.067	[−6.434, 2.239]	0.341
Coping strategies					
Positive coping	19.793	1.564	0.710	[16.709, 22.877]	<0.001
Social support	1.303	1.287	0.055	[−1.235, 3.841]	0.313
Avoidance coping	−2.076	1.344	−0.079	[−4.727, 0.575]	0.124
Work stress	−0.178	0.055	−0.159	[−0.287, −0.069]	0.002

**Note**. Dummy-coded categorical variables were entered using indicator coding. Reference categories were Medical for profession, Permanent for employment status, Hospital for work environment, and >16 years for work experience. B = unstandardized regression coefficient; SE = standard error; β = standardized regression coefficient; CI = confidence interval.

## Data Availability

The data presented in this study are available on request from the corresponding author due to privacy and ethical restrictions.
